# Molecular and Genetic Predictors of Breast-to-Brain Metastasis: Review and Case Presentation

**DOI:** 10.7759/cureus.246

**Published:** 2015-01-31

**Authors:** Zachary Medress, Melanie Hayden Gephart

**Affiliations:** 1 Department of Neurosurgery, Stanford University

**Keywords:** breast cancer, brain metastasis, neurosurgery, oncology, her2/neu

## Abstract

Brain metastases are the most common intracranial malignancy, and breast cancer is the second most common cancer to metastasize to the brain. Intracranial disease is a late manifestation of breast cancer with few effective treatment options, affecting 15-50% of breast cancer patients, depending upon molecular subtype. In this review article, we describe the genetic, molecular, and metabolic changes in breast cancer cells that facilitate breast to brain metastasis. We believe that advances in the understanding of breast to brain metastasis pathogenesis will lead to targeted molecular therapies and to improvements in the ability to prospectively identify patients at increased risk for developing intracranial disease.

## Introduction and background

A significant percentage of patients with breast cancer will acquire brain metastases at some point in their disease, with a significant impact on quality of life and life expectancy. The incidence of brain metastases is between 140,000 and 170,000 cases per year [1]. Breast carcinoma accounts for 12-20% of brain metastases, second only to lung cancer [2]. Autopsy studies have shown brain metastasis in up to 36% of breast cancer patients [3-5] and can involve up to half of patients with certain genetic markers. Breast cancer subtypes include luminal A, luminal B, HER2 positive/non-luminal, and triple negative [6-7]. In patients with breast-to-brain metastasis, HER2 positivity, and luminal-HER2 subtype were significant positive prognostic factors while cerebral progression was the most frequent cause of death [8-9]. Breast cancer brain metastasis is associated with young age, ER negativity [10], and HER-2 overexpression [11-14]. Brain metastasis is a significant cause of morbidity in breast cancer patients, with cognitive impairment detected on neuropsychological testing in up to 67% of patients [15-16]. Current treatment options, frequently used in combination, include surgery, whole-brain radiation therapy, chemotherapy, and stereotactic radiosurgery [17-18]. Without treatment or with corticosteroids alone, median survival of patients with brain metastasis is one and two months, respectively [19-20]. The one-year median survival of patients with brain metastases treated with surgical resection and adjuvant radiosurgery is approximately 50% [21]. As the treatment for systemic breast cancer improves, patients survive longer and the incidence of brain metastases increases.

The development of brain metastases is not random, but rather a coordinated accumulation of opportunistic mutations which enable the breast cancer cells to seed and flourish within the central nervous system (CNS). Successful colonization of distant tissue by tumor cells requires the establishment of a microenvironment in the host tissue that permits cell survival, growth, and invasion. Generally there is a latency of two to three years between surgical removal of primary breast cancer and the appearance of brain metastasis [4], suggesting that tumor cells undergo changes over time that bestow brain tropism. Like other carcinomas that metastasize to the brain, breast cancer has a predilection for brain regions with the highest perfusion, as 80% of metastases occur in the cerebral hemispheres, 15% are located in the cerebellum, and 5% occur in the brainstem [22]. We know breast cancer within the brain is distinct from the primary site: increased Ki67 indices, increased microvascular density, expression of a known pro-metastatic micro-RNAs and gene up-regulation [23-24]. Recently, efforts have been made to understand the genetic and molecular events that predispose cancer to metastasize [25-30], with the goal of prospectively identifying patients at highest risk of developing brain metastasis. 

Consent was formally obtained or waived for all subjects present within this study.

## Review

### HER2-positive breast cancer predisposes to brain metastasis

HER2-positive tumors increase the likelihood of breast-to-brain metastasis or confer enhanced affinity for neural tissue. HER2 overexpression is found in approximately 20% of breast cancers [31-32] and is associated with breast-to-brain metastasis in nearly half of patients with this tumor subtype [5, 33]. Discordance in HER2 status, in which the primary tumor is negative for HER2 while the brain metastasis is HER2-positive, has been found in up to 24% of cases, and this is associated with decreased survival [34-35]. In addition, HER2-positive tumors that are also hormone-receptor-negative have increased risk of relapsing within the CNS [36]. Theories addressing the increased the rate of brain metastasis in HER2-positive breast cancers include homing and tropism of HER2-positive cells in brain parenchyma [37], general aggressiveness of HER2-positive breast cancer and tendency to metastasize to other tissues [38], and increased survival due to improvement in treatment options [39-41]. Molecular therapies that target HER2 include the monoclonal anti-HER2 antibody trastuzumab (Herceptin) and pertuzumab (Perjeta) (Genentech, South San Francisco, CA), and tyrosine kinase inhibitors, such as lapatinib (Tykerb) (GlaxoSmithKline, Middlesex, UK) [42]. As in primary breast cancer, it is hypothesized that trastuzumab functions by triggering the internalization and degradation of HER2 through the action of c-Cbl, a tyrosine kinase-ubiquitin ligase [43-44]. Pertuzumab is a monoclonal antibody that inhibits the dimerization of HER2 [45]. Lapatinib is a dual tyrosine kinase inhibitor that acts on both HER1 and HER2 by reversibly inhibiting the ATP-biding site of the tyrosine kinase domains of the HER receptors [46]. The proportion of patients with metastatic HER2-positive breast cancer who demonstrate a clinical response to trastuzumab, defined as at least a >50% decrease in tumor volume, is up to 34% [47].

HER2 expression has direct links to tumor biology that enhances growth in the brain. When HER2 is overexpressed in breast cancer cell lines, TGFβ production is increased, leading to activation of TGF/SMAD pathways and expression of transcriptional HER2 of E-cadherin, including SNAIL, SLUG, and ZEB-1. Inhibition of HER2 by cucurbitacin B leads to suppression of brain metastasis *in vivo *[48]. These findings support the notion that HER2 contributes to epithelial to mesenchymal transition (EMT) and breast-to-brain metastasis through the production of TGFβ, a known master regulator of EMT [49-51]. Given that HER2-positive tumors found to co-express SNAIL did not respond to trastuzumab therapy [52], expression of TGF dependent proteins may further contribute to trastuzumab resistance and EMT [53]. Breast cancer is the most common solid tumor to metastasize to the leptomeninges [54], and the incidence of leptomeningeal metastasis is higher in HER2-positive tumors compared to ER-positive lobular and triple negative breast cancer [5, 55-56]. HER2-positive tumors remain a difficult subtype to treat, given the marked predisposition for difficult to treat brain metastasis. 

### Brain endothelial cells interact with breast cancer metastases

The blood-brain barrier (BBB) poses a significant obstacle to infiltrating tumor cells via tight junctions, junctional adhesion molecules, and astrocyte foot processes [57-58]. Real-time imaging of metastasizing cancer cells *in vivo *has shown that brain metastasis is an inefficient process in which cells undergo high rates of attrition, and that early extravasation and persistent close contact with endothelial cells are critical features [59]. MRI studies of injected tumor cells show that approximately 1.5% of injected cells form metastases in the brain [60]. Examination of early micrometastases in the brain shows that 95% of metastatic cells grow along vessels as opposed to isolated colonies within the brain parenchyma [61], suggesting that vascular basement membrane may represent an important “soil” that facilitates brain metastasis [62].

Endothelial cells may help breast cancer enter the CNS. Breast cancer cell transmigration is augmented by human brain endothelial cells as endothelial cell expression of COX-2 induces expression of matrix metalloproteinases in cancer cells [63]. αB-crystallin, a molecular chaperone primarily expressed in triple negative breast cancer, is associated with poor prognosis. When overexpressed, metastatic breast cancer cells exhibit enhanced adhesion to human brain microvascular endothelial cells [64]. JAM-A, a component of the endothelial tight junction complex, is highly expressed in normal mammary epithelium, yet down-regulated in breast cancer cells in the brain [65]. JAM-A expression positivity is correlated with a poor prognosis [66]. Cathepsin S, a protein that mediates transmigration of tumor cells across the BBB via proteolytic processing of JAM, was independently associated with breast-to-brain metastasis [67]. A study involving the injection of human estrogen receptor (ER)-negative pleural malignant breast cancer cells intra-arterially in rats to select for brain tropism, revealed COX2, the EGFR ligand HBEGF, and the alpha2,6-sialyltransferase ST6GALNAC5 as important mediators of brain metastasis. In particular, ST6GALNAC5 was found to mediate adhesion of tumor cells to brain endothelial cells and subsequent entry through the blood brain barrier [68]. Breast cancer cells metastatic to the brain have refined a specific capacity to interact favorably with CNS endothelial cells. 

### Breast cancer adapts to the brain microenvironment

Astrocytes play an important role in the survival of breast cancer upon entering the brain. Once through the BBB, invading breast cancer cells are surrounded by reactive astrocytes, quickly localizing to tumor cells through the up-regulation of matrix metalloproteinase-9 [69]. Astrocytes have been shown to secrete matrix metalloproteinases (MMP), including MMP-2 and MMP-9, and that culturing metastatic breast cancer cells with astrocyte-conditioned media lead to increased invasive ability [70]. Reactive astrocytes defend against metastatic invasion by up-regulating plasmin, which leads to the secretion of the paracrine death signal FasL and inactivates L1CAM, a molecule used by metastatic tumor cells to disperse along capillaries. To combat this onslaught, metastatic breast cancer cells highly express serpins, anti-plasminogen activators, including neuroserpin and serpin B2 [59]. Reactive glia may also inadvertently support the growth of metastatic tumor cells. Co-culture experiments of tumor cells and glia resulted in a five-fold increase in tumor cell proliferation [59]. When co-cultured with murine astrocytes, breast cancer cells up-regulated survival genes, including GSTA5, BCL2L1, and TWIST1, and the degree of up-regulation correlated with resistance to chemotherapy [71]. In fact, astrocytes may protect cancer cells from chemotherapeutic agents [72]. Breast cancer stem cell expression of IL-1B leads to astrocyte activation and expression of JAG1 in astrocytes, which leads to increased Notch signaling in cancer stem cells and represents an important self-renewal pathway in metastatic tumor cells [73]. Adaptive strategies by breast cancer cells and reaction of astrocytes to breast cancer cells may facilitate breast tumor cell survival in the brain.

Gene expression profiling performed over time as breast cancer cells underwent progressive invasion of brain tissue reveals a transient activation of genes involved in homeostasis and stress, and then activation of genes involved in cell division and morphology as cells were faced with surviving in a new microenvironment [74]. These experiments showed that breast cancer cells co-cultured with brain tissue express many brain-specific genes, likely due to the effect of secreted signals [75].

Breast tumor metabolism within the brain niche enables cancer growth and survival. Despite the high energetic demand of neural tissue, glucose levels in the interstitial space of the brain are highly regulated by astrocytes and lower than blood glucose levels [76-77], suggesting that breast cancer cells must undergo metabolic reprogramming in order to thrive in the brain interstitial microenvironment. Breast-to-brain metastasis demonstrates enhanced ability to survive independently of glucose metabolism by undergoing high rates of gluconeogenesis and oxidation of glutamine and branched chain amino acids. Silencing of fructose-1,6-bisphosphatase, an essential component of gluconeogenesis, leads to improved survival in mice bearing orthotopic breast-to-brain metastasis [78].

### Case presentation

**Figure 1:**
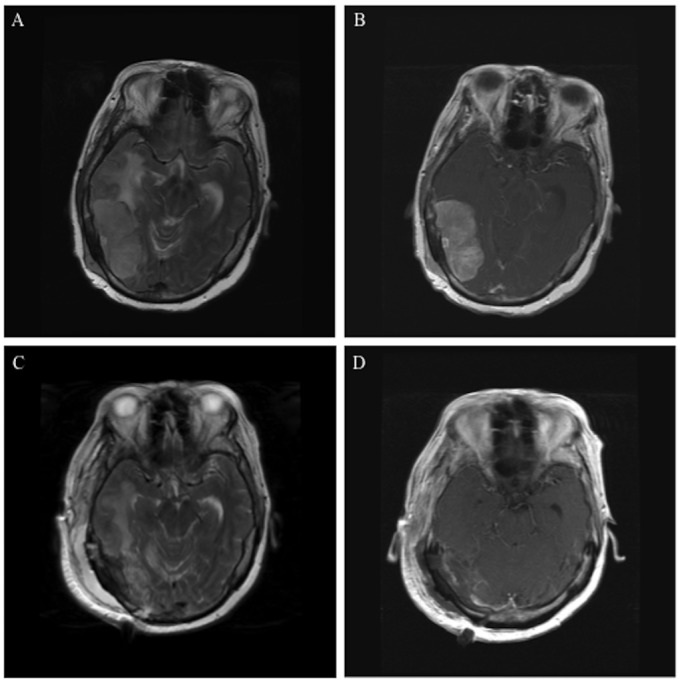
Resection of Dural-based Brain Metastasis Pre- and postoperative MRI images demonstrate resection of a breast cancer dural-based metastasis. Preoperative axial T2 (A) and T1 with contrast images (B) show a heterogeneously enhancing 2.9 x 5.9 x 2.9 cm dural-based tumor in the right temporal-parietal region associated with significant peri-tumoral edema and midline shift. Postoperative T2 (C) and T1 with contrast images (D) demonstrate tumor resection.

A 59-year-old woman with a five-year history of invasive ductal ER, PR, and HER2/neu-positive breast cancer presented with a history of headache, nausea, and decreased oral intake. The patient had undergone a mastectomy, followed by neoadjuvant chemotherapy (doxorubicin, cyclophosphamide, paclitaxel, trastuzumab, paclitaxel), and radiation. On initial examination, she had decreased orientation and level of consciousness, hemineglect, and left-sided pronator drift. A head CT demonstrated a large right-sided temporal-parietal mass measuring 2.9 x 5.9 x 2.9 cm with surrounding edema, 10 mm of midline shift, and subfalcine herniation. A brain MRI re-demonstrated a large, right-sided, heterogeneously enhancing, dural-based, temporal-parietal mass (Figures 1A, 1B).

Given the progressive nature of the patient’s symptoms from the large dural-based mass causing midline shift, subfalcine herniation, and significant peritumoral edema, it was recommended that the patient undergo a craniotomy for tumor resection. The craniotomy was performed with care to maximize control of the transverse sinus. The tumor was devascularized from its base with meticulous hemostasis and microsurgical dissection between the edematous brain and the tumor capsule.

The patient tolerated the operation well. Postoperatively, the patient was immediately more responsive, pronator drift resolved, and she was referred for external beam radiation. Postoperative MRI confirmed excellent tumor resection (Figures 1C, 1D). Pathologic analysis showed metastatic carcinoma from a breast primary tumor. Abundant mitotic figures and foci of necrosis were evident within the tumor. Immunohistochemical analysis showed that the tumor was ER-positive, PR-negative, and HER2/neu-positive.

## Conclusions

Brain metastasis is a common but late complication of breast cancer that contributes to significant morbidity and mortality. Patients with HER2-positive breast cancer are at increased risk of developing breast-to-brain metastasis, and inhibition of HER2 in experimental models has led to the suppression of brain metastasis. Recent work has demonstrated the genetic, molecular, and metabolic changes that occur in breast cancer cells as they gain the ability to survive in the brain microenvironment. We believe that further advances in the understanding of the pathogenesis of breast to brain metastasis will lead to improved targeting of brain lesions and the ability to prospectively identify breast cancer patients at highest risk of developing intracranial disease.
